# Stratification of Diversity and Activity of Methanogenic and Methanotrophic Microorganisms in a Nitrogen-Fertilized Italian Paddy Soil

**DOI:** 10.3389/fmicb.2017.02127

**Published:** 2017-11-13

**Authors:** Annika Vaksmaa, Theo A. van Alen, Katharina F. Ettwig, Elisabetta Lupotto, Giampiero Valè, Mike S. M. Jetten, Claudia Lüke

**Affiliations:** ^1^Department of Microbiology – Institute of Water and Wetland Research, Radboud University, Nijmegen, Netherlands; ^2^Research Centre for Food and Nutrition, Consiglio per la Ricerca in Agricoltura e l’Analisi dell’Economia Agraria, Rome, Italy; ^3^Research Centre for Cereal and Industrial Crops, Consiglio per la Ricerca in Agricoltura e l’Analisi dell’Economia Agraria, Vercelli, Italy

**Keywords:** anaerobic oxidation of methane, paddy fields, 16S rRNA gene amplicon sequencing, *Methanoperedens nitroreducens*, NC10 phylum bacteria, *Bathyarchaeota*

## Abstract

Paddy fields are important ecosystems, as rice is the primary food source for about half of the world’s population. Paddy fields are impacted by nitrogen fertilization and are a major anthropogenic source of methane. Microbial diversity and methane metabolism were investigated in the upper 60 cm of a paddy soil by qPCR, 16S rRNA gene amplicon sequencing and anoxic ^13^C-CH_4_ turnover with a suite of electron acceptors. The bacterial community consisted mainly of *Acidobacteria, Chloroflexi, Proteobacteria, Planctomycetes,* and *Actinobacteria*. Among archaea, *Euryarchaeota* and *Bathyarchaeota* dominated over *Thaumarchaeota* in the upper 30 cm of the soil. *Bathyarchaeota* constituted up to 45% of the total archaeal reads in the top 5 cm. In the methanogenic community, *Methanosaeta* were generally more abundant than the versatile *Methanosarcina.* The measured maximum methane production rate was 444 nmol g_dw_h^-1^, and the maximum rates of nitrate-, nitrite-, and iron-dependent anaerobic oxidation of methane (AOM) were 57 nmol, 55 nmol, and 56 nmol g_dw_h^-1^, respectively, at different depths. qPCR revealed a higher abundance of ‘*Candidatus* Methanoperedens nitroreducens’ than methanotrophic NC10 phylum bacteria at all depths, except at 60 cm. These results demonstrate that there is substantial potential for AOM in fertilized paddy fields, with ‘*Candidatus* Methanoperedens nitroreducens’ archaea as a potential important contributor.

## Introduction

Methane, a significant greenhouse gas, has up to 34 times the global warming potential over 100 years compared to carbon dioxide (Myhre et al., 2013). Paddy fields contribute substantially to atmospheric methane concentrations and release 25–300 Tg of CH_4_ per annum (Bridgham et al., 2013), representing 10–20% of global methane emissions (Conrad, 2009; Bodelier, 2011). In the next decades, the land area designated for rice cultivation is predicted to increase even further. Without mitigation measures, this will result in elevated methane emission to the atmosphere.

The microbial community structure of paddy fields is influenced by several environmental and anthropogenic factors. Alteration in microbial community composition in paddy fields have been studied with respect to flooding (Rui et al., 2009), fertilization and straw application (Bao et al., 2016), temperature (Conrad et al., 2009; Noll et al., 2010), rice cultivar and soil type (Conrad et al., 2008), and plant growth stage (Breidenbach and Conrad, 2015). Paddy fields provide a habitat for both aerobic and anaerobic methanotrophs. Aerobic methanotrophs are found in the oxic layers of the soil and in oxic microhabitats of the rhizosphere. Methanogenic archaea, anaerobic methanotrophic archaea and/or bacteria thrive preferentially in the anoxic compartments of the waterlogged soil. The flux of methane to the atmosphere is the net result of production and consumption by methanogenic and methanotrophic microorganisms.

Since the discovery of “*Bacillus methanicus”* (Söhngen, 1906), aerobic methane-oxidizing bacteria (MOB) have been extensively studied. MOBs were long considered the only microbes capable of oxidizing methane. Currently, MOB belong to the phyla *Proteobacteria* and *Verrucomicrobia* (Op den Camp et al., 2009; Semrau et al., 2010). Proteobacterial aerobic methanotrophs inhabit a wide variety of environments, ranging from tundra soil (Dedysh et al., 2004) and arctic permafrost (Liebner et al., 2009) to sewage treatment sludge (Ho et al., 2013). Phylogenetic analyses of both 16S rRNA and the particulate methane mono-oxygenase subunit A (*pmoA*) gene have classified *Proteobacteria* into *Gammaproteobacteria* (Type I methanotrophs) and *Alphaproteobacteria* (Type II methanotrophs) (Trotsenko and Murrell, 2008; Semrau et al., 2010). Type I methanotrophs belong to the genera *Methylosarcina, Methylobacter, Methylomonas, Methylomicrobium, Methylosoma, Methylosphera,* and *Methylovulum* (Type Ia) and *Methylococcus, Methylocaldum, Methylogaea, Methylohalobius,* and *Methylothermus* (Type Ib). Alphaproteobacterial MOB belong to the genera *Methylocystis* and *Methylosinus* (Type IIa) and the genera *Methylocella, Methylocapsa,* and *Methyloferula* (Type IIb) (Dumont et al., 2014; Zheng et al., 2014; Knief, 2015). Aerobic methanotrophs have been detected in several paddy field soils (Ho et al., 2011; Lüke and Frenzel, 2011; Lee et al., 2014), and furthermore, it has been suggested that Type I methanotrophs can likely outcompete Type II methanotrophs for substrates in these nitrogen-loaded environments (Zheng et al., 2014). Compared to the proteobacterial aerobic methanotrophs, the more recently discovered *Verrucomicrobia* often inhabit more extreme environments with low pH values and/or high temperatures (Dunfield et al., 2007; Op den Camp et al., 2009; Sharp et al., 2014; van Teeseling et al., 2014).

Rice cultivation under waterlogged conditions creates anoxia in the majority of soil compartments and, consequently, provides a suitable habitat for methanogenic microorganisms. Rice maturation with the developed and decaying rhizosphere, releases root exudates that, together with dead roots, provide organic matter for an anaerobic food chain. Oxygen influx to soil occurs through diffusional transport via the aerenchyma and radial oxygen loss of the rice roots (Armstrong, 1971; Li and Wang, 2013). Although traditionally considered strict anaerobes, methanogens have been detected in the rhizosphere and on rice roots in several studies (Chin et al., 2004; Xu et al., 2012; Edwards et al., 2015; Lee et al., 2015). Lee et al. (2015) observed a higher abundance of methanogens in the rhizosphere than in bulk soil (Lee et al., 2015). The methanogens in the rhizosphere may live in non-active roots where no oxygen is released or, alternatively, may be oxygen tolerant and have mechanisms to counteract reactive oxygen radical species, as investigated for Rice Cluster I (RC I) (now known as *Methanocella*) methanogens (Erkel et al., 2006). The genomes of RC I harbor genes encoding catalase, three different superoxide anion scavengers, superoxide dismutase and two different super oxide reductase genes for oxygen detoxification (Erkel et al., 2006). The up-regulation of catalase genes in response to oxygen exposure has been observed in both *Methanosarcina* and *Methanocella* (Angel et al., 2011).

Both acetoclastic and hydrogenotrophic methanogens have been identified in paddy fields. Methanogenic archaea of the order *Methanosarcinales* derive methane from the methyl group of compounds such as methanol and methylamine, and until now, only *Methanosarcina* and *Methanosaeta* are known to use acetate for methane production (Jetten et al., 1992; Costa and Leigh, 2014; Welte and Deppenmeier, 2014). Hydrogenotrophic methanogens belonging to the orders *Methanomicrobiales*, *Methanobacteriales* and *Methanocellales* have been commonly found in paddy fields, with the exception of *Methanococcales,* which barely have been detected (Watanabe et al., 2010; Lee et al., 2015). Many of these hydrogenotrophic methanogens can use formate as a substrate but are unable to utilize acetate. Archaea belonging to RC I (*Methanocella*) (Kögel-Knabner et al., 2010), which forms a separate phylogenetic lineage branching between the orders *Methanosarcinales* and *Methanomicrobiales*, are considered key methanogens in rice fields. The reaction stoichiometry of methanogenesis (Conrad and Klose, 1999) indicates that acetoclastic methanogens could contribute approximately two-thirds to methane production, consistent with the dominance of acetoclastic over hydrogenotrophic methanogenesis in paddy fields (Krüger et al., 2001).

Previous theories suggesting a decrease in methane flux as a result of direct stimulation of methanotrophs after amendment with nitrogen fertilizers were unable to link observations to the activity of the denitrifying anaerobic methanotrophic bacteria and archaea as these microorganisms, were discovered only recently compared to the aerobic methanotrophs. Nitrite- and nitrate-dependent anaerobic oxidation of methane (AOM) were first described in 2006 in an enrichment culture consisting of archaea distantly related to ANME-2d and of bacteria that consume nitrite as an electron acceptor to oxidize methane anaerobically (Raghoebarsing et al., 2006). This novel denitrifying, methanotrophic bacterium of the candidate division NC10 was named *‘Candidatus* Methylomirabilis oxyfera’ (Ettwig et al., 2010). Despite its preference for an anoxic habitat, it is postulated to have an intra-aerobic metabolism. The genome of the bacterium contains all genes of the aerobic methanotrophic pathway and encodes a particulate methane mono-oxygenase complex that can use the O_2_ released from nitric oxide for methane oxidation, similar to aerobic methanotrophs (Ettwig et al., 2010).

The genome of ANME-2d archaea was sequenced in 2013 and responsible organism named ‘*Candidatus* Methanoperedens nitroreducens’ (Haroon et al., 2013). This nitrate-reducing archaeon employs a reverse methanogenesis pathway to oxidize methane. The genomes of three different strains of ‘*Candidatus* Methanoperedens nitroreducens’ have been published, and the necessary genes for nitrate reduction and the methanogenic pathway have been identified (Haroon et al., 2013; Arshad et al., 2015; Vaksmaa et al., 2017a,b). Nitrite- and nitrate-dependent AOM microorganisms and/or activity have been detected in several freshwater environments, including paddy fields (Vaksmaa et al., 2016; Welte et al., 2016). Recently it was demonstrated that ‘*Candidatus* Methanoperedens nitroreducens’ can also oxidize methane using iron as electron acceptor (Ettwig et al., 2016).

Besides so far know methanotrophs and methanogens, recent investigations of microbial “dark matter” discovered key genes of the methane pathway to be present in phyla, which previously were not linked to the ability to produce or consume methane. Phylum *Bathyarchaeota,* renamed from *Miscellaneous Crenarchaeotic Group* is a deeply branching phylum consisting of 17 sub-groups (Kubo et al., 2012). It is abundant in marine environments but is also found in extreme habitats like hot springs, cold sulfur springs, Polar Regions and in mesophilic habitats like sewage waste, fresh water lakes and paddy fields. Though there are no pure isolates, based on culture independent methods, their function was speculated to be important in the global cycle of carbon (Parkes et al., 2005). To date there are eight different genomes annotated, out of which two BA1 and BA2 are hypothesized to be methane metabolizers (Evans et al., 2015; He et al., 2016).

Majority of previous studies of paddy field microbial communities have focused on either a specific group of microorganisms or environmental or anthropogenic effect on methane emissions or sampling had been carried out at a single depth, hindering direct comparison. The aim of the present study was to explore how the microbial communities in a paddy field are influenced by spatial factors along a depth gradient. The objectives of this study were (i) to characterize the bacterial and archaeal communities in a paddy field soil core by 16S rRNA gene amplicon sequencing with a focus on methane cycle-related organisms; (ii) determine the abundances of total bacteria, total archaea, ‘*Candidatus* Methanoperedens nitroreducens,’ NC10 phylum bacteria and *Bathyarchaeota*; and (iii) estimate the anaerobic methane oxidation potential using nitrate, nitrite and iron as electron acceptors at different soil depths.

## Materials and Methods

### Soil Sampling

Paddy field soil cores were sampled in August 2015 at the Italian Rice Research Unit in Vercelli, Italy (08°22’25.89”E; 45°19’26.98”N). The sampling fields were cultivated with the rice variety *Oryza sativa* temperate japonica Onice. The paddy fields were flooded for about 90 days, with fertilizer applied in April and twice in June. Soil cores were sampled in triplicate with 80-cm soil augers at approximately 5-m intervals. The porewater nitrate and ammonium concentrations were in average 0.6 μM and 6.8 μM throughout the 80 cm. Amorphous iron oxides over a 50 cm core were in top 25 cm in average 28.5 μmol per gram wet weight (g_ww_) soil and in lower 25 cm 54.8 μmol per g_ww_ soil, with one maxima at 11 cm 68.6 μmol per g_ww_ soil and at 31 cm 76.0 μmol per g_ww_ soil (data obtained from the previous year), analysis was performed as described in Egger et al. (2015). For AOM and methanogenic activity incubation assays, the soil was sliced in the field and placed immediately in anaerobic jars. For DNA extraction, the samples were stored in 50-ml conical centrifuge tubes. All samples were stored at 4°C at the field site laboratory until transport on cool compresses by car. After transport to the lab, samples for DNA extraction were immediately frozen at -20°C, and samples for activity experiments were stored at 4°C.

### Methane Measurements

To measure methane entrapped in the soil, three separate cores with lengths of 51, 58, and 68 cm were sampled. Immediately after sampling, while releasing the core from the auger, samples were taken with a 5-ml open-end syringe. These samples were then transferred to pre-weighed 120-ml bottles filled with saturated NaCl solution. The bottles were sealed with screw-caps with rubber stoppers. The CH_4_ concentration was quantified by gas chromatography (Hewlett Packard 5890, United States). Methane concentrations were calculated per gram dry weight (g_dw_) of the sampled soil at the respective depth.

### DNA Extraction

For DNA extraction soil cores were divided to 13 different depths. Soil from the same depth of three cores was pooled. DNA was extracted from approximately 0.25 g of soil in duplicate using a PowerSoil DNA isolation Kit (MO BIO Laboratories Inc., Carlsbad, CA, United States) according to the manufacturer’s protocol. DNA was extracted from the following depths: 0 cm, 2.5 cm, 5 cm, 7.5 cm, 10 cm, 15 cm, 20 cm, 25 cm, 30 cm, 35 cm, 40 cm, 50 cm, and 60 cm. DNA quantity and quality were assessed by UV-VIS spectroscopy (NanoDrop, ND-1000, Isogen Life Science, Netherlands).

### Quantification by qPCR

Quantification of the total bacterial and total archaeal communities using the 16S rRNA gene was performed in triplicate using the duplicate DNA extractions from each depth sample described above. For archaea, the following primers were used: forward Arch-349 (5′GYGCASCAGKCGMGAAW3′) (Takai and Horikoshi, 2000) and reverse Arch-807 (5′GGACTACVSGGGTATCTAAT3′) (Wang and Qian, 2009). For bacteria, the primers were forward Bact-341 (5′CCTACGGGNGGCWGCAG3′) and reverse Bact-785 (5′GACTACHVGGGTATCTAATCC3′) (Herlemann et al., 2011). Bathyarchaeota were targeted by primers amplifying 16S rRNA gene: MCG528 forward and MCG732 reverse (Kubo et al., 2012). ‘*Candidatus* Methanoperedens nitroreducens’ was targeted by primers amplifying the *mcrA* gene: McrA159F forward and McrA345R reverse (Vaksmaa et al., 2017a). The 16S rRNA gene of the NC10 phylum was amplified with the primers p2F_DAMO (5′GGGGAACTGCCAGCGTCAAG3′) and p2R_DAMO (5′CTCAGCGACTTCGAGTACAG3′) (Ettwig et al., 2009). All qPCR reactions were performed using PerfCTa Quanta master mix (Quanta Biosciences, United States) and 96-well optical plates (Bio-Rad, United States) on a Bio-Rad CFX96 Real-Time C1000 Touch Thermal Cycler (Bio-Rad, United States), as described in Vaksmaa et al. (2016, 2017a). Absolute quantification was performed by comparison to standard curves obtained using a 10-fold serial dilution of pGEM-T Easy plasmid DNA (Promega, United States) with an insert of the target gene obtained using the same primers as used for qPCR. Standard curve samples were used as a control for each qPCR run.

### Amplicon Sequencing

The following primers were used for 16S rRNA gene amplification: forward Arch-0349 and reverse Arch-807 for archaea and forward Bact-0341 and reverse Bact-785 for bacteria. The amplicons were generated in a two-step reaction. DNA was pooled in equimolar amounts per depth to perform PCR under the following conditions: initial denaturation at 96°C for 3 min; 30–35 cycles of denaturation at 96°C for 40 s, primer annealing at 60°C (for archaea) or 61°C (for bacteria) for 30 s, and elongation at 72°C for 40 s; and a final elongation at 72°C for 2 min. Each PCR product was verified by 1% gel electrophoresis. The obtained PCR products were purified with a GeneJet PCR purification kit (Thermo Scientific, Netherlands). A second PCR was then performed with the same primers described above, which were extended with adapter sequences, specific barcodes and key sequences compatible with Ion Torrent sequencing at the 5′ end. The reaction conditions for this PCR were an initial denaturation at 96°C for 10 min; 10 cycles of denaturation at 96°C for 1 min, primer annealing at 60°C or 61°C for 1 min and elongation at 72°C for 2 min; and a final elongation step at 72°C for 10 min. The products were again pooled per depth and purified as described above. The DNA concentrations of the purified PCR products were then measured and diluted to a range of 0.2–0.4 ng/μl. The concentrations and fragment lengths of the libraries were determined with a Bioanalyzer 2100 and a High Sensitivity DNA kit (Agilent Technologies, United States). The obtained libraries were diluted to a final concentration of 100 pM, and the different barcoded libraries were pooled in equimolar amounts before sequencing. For Ion Torrent sequencing, the library fragments were attached to Ion Sphere particles using an Ion One Touch Instrument and Ion PGM Template OT2 400 Kit (Life Technologies, United States) according to the manufacturer’s instructions. After enrichment of the template-positive Ion Sphere Particles using the Ion One Touch ES (Life Technologies, United States), the samples were loaded on an Ion 316 v2 Chip. The DNA fragments were then sequenced using the Ion PGM Sequencing 400 Kit and 850 nucleotide flows according to the manufacturer’s instructions.

### Analysis of 16S rRNA Gene Amplicon Data

The raw sequencing reads were automatically separated into clusters of each depth based on the unique barcodes. After sequencing, all raw reads were imported into CLC Genomics Workbench vs. 9 (QIAGEN Aarhus A/S, Denmark) for initial data analysis, including trimming of low-quality and short reads (cut-off value 200 nucleotides). After trimming, 6,661–11,785 reads were obtained per corresponding depth for archaea; the number of reads obtained per depth for bacteria was 4,477–7,198 reads. The exported reads were further processed using the automated pipeline of Silva NGS (Silva Next Generation Sequencing) of the SILVA rRNA gene database project (SILVAngs 1.2) (Quast et al., 2013). In this process, each read was aligned using the SILVA Incremental Aligner [SINA v1.2.10 for ARB SVN (revision 21008)] (Pruesse et al., 2012) against the SILVA SSUrRNA SEED and quality controlled (Quast et al., 2013). Reads shorter than 50 aligned nucleotides and reads with more than 2% ambiguities or 2% homopolymers were excluded from further processing. Putative contaminants, artifacts and reads with low alignment quality (50 alignment identity, 40 alignment score reported by SINA) were identified and excluded from downstream analysis. After these initial quality control steps, identical reads were identified (dereplication), unique reads were clustered (OTUs) on a per sample basis, and the reference read of each OTU was classified. Dereplication and clustering were performed using cd-hit-est (version 3.1.2^1^) (Li and Godzik, 2006) running in accurate mode, ignoring overhangs, and applying identity criteria of 1.00 and 0.98, respectively. Classification was performed by local nucleotide BLAST search against the non-redundant version of the SILVA SSU Ref dataset (release 119^2^) using blastn^3^ (version 2.2.28+) with standard settings (Camacho et al., 2009). The classification of each OTU reference read was mapped onto all reads that were assigned to the respective OTU. This mapping yielded semi-quantitative information (number of individual reads per taxonomic path), within the limitations of PCR and sequencing technique biases, and multiple rRNA operons. Reads without any BLAST hits or reads with weak BLAST hits, in which the function ∖(% sequence identity + % alignment coverage)/2′′ did not exceed a value of 93, remained unclassified. These reads were assigned to the metagroup∖No Relative” in the SILVAngs fingerprint and Krona charts (Ondov et al., 2011). This method was first used in the publications (Ionescu et al., 2012; Klindworth et al., 2013). The amplicon sequencing data were deposited to the Short Read Archive under Bioproject ID PRJNA378333. Estimated quantities of individual taxa were calculated by multiplication of relative amplicon sequence data with qPCR data.

### Soil Incubations

Soil samples from the three cores were pooled at depths of 0–5 cm, 5–10 cm, 10–20 cm, 20–30 cm, 30–40 cm, 40–50 cm, and 50–60 cm. Soil slurries for each depth were prepared by mixing the soil with mineral salt medium as described by Ettwig et al. (2008). Activity assays were performed in 120-ml serum bottles with 60 ml of soil slurry. The wet and dry soil weight ratio of the slurry was determined in duplicate at each depth. The incubation bottles were sealed with red butyl rubber stoppers and crimp-caps. The headspace was exchanged with Ar/CO_2_ by five cycles of vacuum and gassing, with a final overpressure of 0.5 bar. Treatments at each of the depths were performed in duplicate and consisted of adding 5 mM NaNO_3_, 1 mM NaNO_2_, 20 mM iron nitrilotriacetic acid (FeNTA), or 20 mM ferrihydrite with 10% ^13^C-CH_4_ v/v (final concentrations) and controls in which either 10% CH_4_ v/v was added or no additions were made to the soil slurry. Each treatment was performed in duplicate with triplicate headspace measurements to quantify the CH_4_ concentration by gas chromatography (Hewlett Packard 5890, United States) as described previously (Ettwig et al., 2009). Headspace measurements were carried out over the period of 118 days, with methane concentration measured at day 0, 7, 14, 21, 46, 54, 85, 98, and 118 and the net production or consumption rates were calculated during the linear phase.

## Results

### Methane Measurements in the Soil Core

**FIGURE 1:**
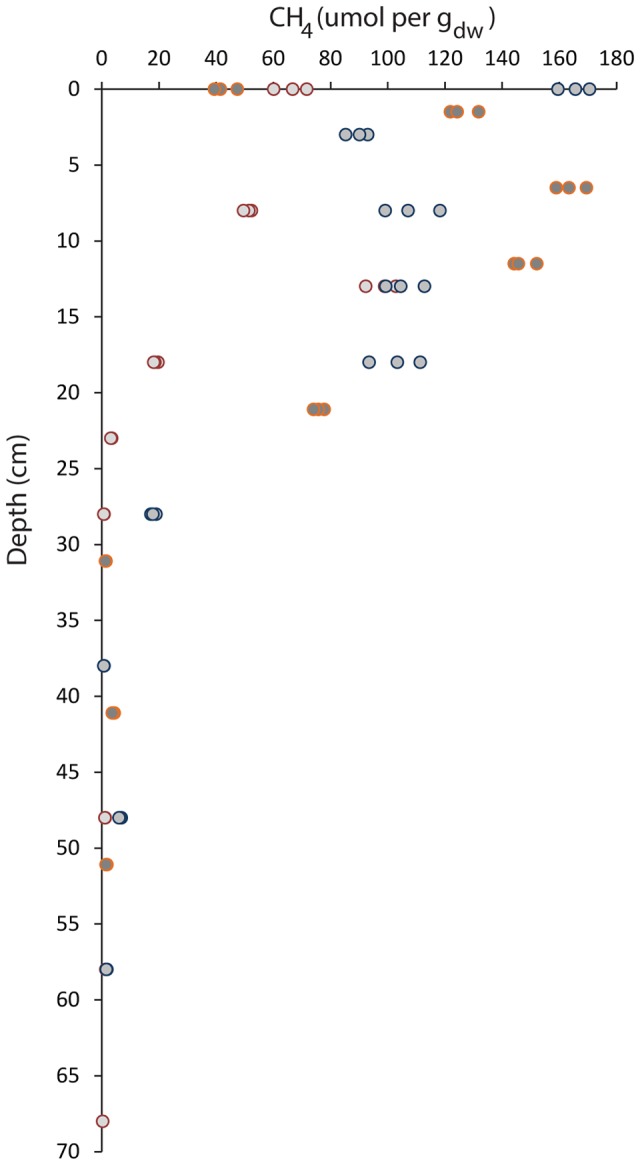
Vertical profile of methane concentrations along the depth gradient of the paddy field soil. The soil depth is depicted vertically, and the methane concentration in μmol per g_dw_ is depicted horizontally. Three separate cores were sampled up to the maximum depth of 68 cm. Measurements of each sample were performed in triplicate by gas chromatography.

The highest methane concentration was measured in the top 15 cm of the soil (**Figure 1**). The highest peak was measured at 0 cm and 6.5 cm and corresponded to a methane concentration of approximately 165 μmol per g_dw_ in two of the three cores. Below 15 cm, a rapid decrease in the methane concentration was observed; at a depth of 28 cm, methane concentrations were less than 7 μmol per g_dw_. At depths of 50 cm and below, the methane concentration was at the detection limit of 0.4–2 μmol per g_dw_.

### Quantification of Total Bacteria, Archaea, and Subgroups of Known Anaerobic Methanotrophs

**FIGURE 2:**
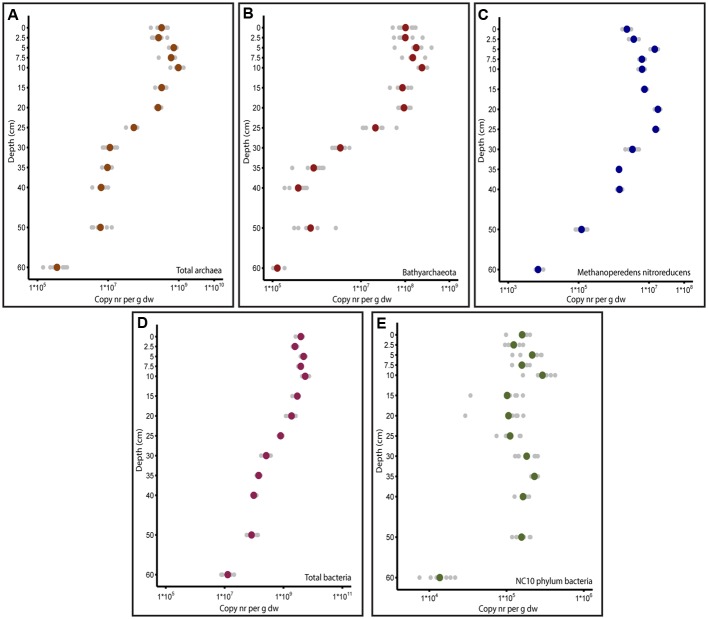
Depth profile of copy numbers of genes of interest obtained by qPCR. In all figures, the soil depth is depicted vertically. Horizontally, the copy numbers obtained by qPCR per gram dry weight are presented in log scale. **(A)** 16S rRNA gene copy numbers of total archaea amplified with Arch349F/Arch807R primers. **(B)** 16S rRNA gene copy numbers of *Bathyarchaeota* amplified with MCG528F/MCG732R primers. **(C)** *mcrA* gene copy numbers of Methanoperedens nitroreducens quantified with McrA159F/McrA345R primers. **(D)** 16S rRNA gene copy numbers of total bacteria quantified with Bac341F/Bac785R primers. **(E)** 16S rRNA gene copy numbers of NC10 phylum bacteria quantified with p2F_DAMO/p2R_DAMO primers.

The total abundance of bacteria, archaea and nitrate- and nitrite dependent anaerobic methanotrophs was quantified by qPCR. The total bacterial abundance was higher than the archaeal abundance at all depths of the soil core. As depicted in **Figure 2**, the highest copy number obtained with the archaeal primer combination was observed at a depth of 10 cm (1.0 ± 0.3^∗^10^9^ 16S rRNA gene copies per g_dw_). Below a depth of 20 cm (2.6 ± 0.4^∗^10^8^ 16S rRNA gene copies per g_dw_), the archaeal copy numbers decreased gradually until 60 cm, where 4.1 ± 2.2^∗^10^5^ 16S rRNA gene copies per g_dw_ was observed_._ The highest amount of bacterial copies was observed at a depth of 10 cm (5.6 ± 1.4^∗^10^9^ 16S rRNA gene copies per g_dw_), and the lowest number was observed at a depth of 60 cm (1.4 ± 0.6^∗^10^7^ 16S rRNA gene copies per g_dw_). The known archaeal methanotroph ‘*Candidatus* Methanoperedens nitroreducens’ exhibited the highest abundance at 20 cm, with 1.8 ± 0.3^∗^10^7^ *mcrA* gene copies per g_dw_, and lowest abundance at 60 cm, with 7.2 ± 1.5^∗^10^3^ *mcrA* gene copies per g_dw_. The anaerobic methanotrophs belonging to NC10 phylum bacteria had two maxima at depths of 10 cm (2.3 ± 0.7^∗^10^5^ 16S rRNA gene copies per g_dw_) and 35 cm (2.3 ± 0.2^∗^10^5^ 16S rRNA gene copies per g_dw_). The lowest abundance was observed at a depth of 60 cm (1.5 ± 0.5^∗^10^4^ 16S rRNA gene copies per g_dw_). Among the targeted anaerobic methanotrophs, ‘*Candidatus* Methanoperedens nitroreducens’ had higher gene copy numbers than NC10 phylum bacteria at all depths except 60 cm, where NC10 phylum bacteria outnumbered ‘*Candidatus* Methanoperedens nitroreducens.’

### Amplicon Sequencing of the 16S rRNA Gene in the Bacterial Community

**FIGURE 3:**
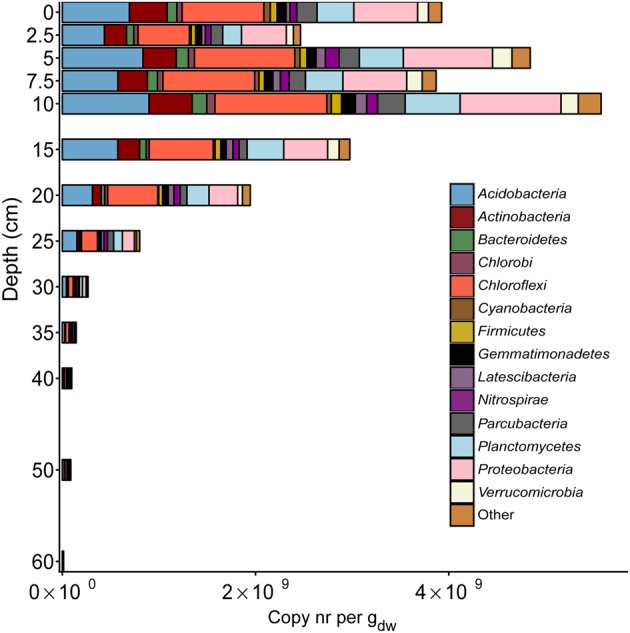
Distribution of 16S rRNA gene reads of major bacterial phyla along the depth profile of the paddy soil core. The soil depth in centimeters is depicted vertically, whereas the total amount of 16S rRNA gene amplicons per gram dry weight is depicted horizontally. The colored bars represent the relative amount of gene copies matching a bacterial phylum present in the soil at a particular depth.

**FIGURE 4:**
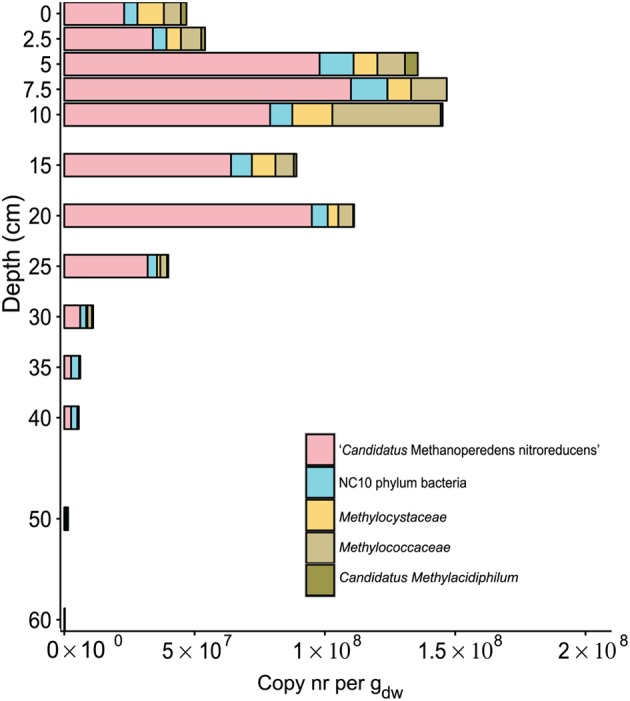
Distribution of sequence reads of proteobacterial (*Methylococcaceae*, *Methylocystaceae*) and verrucomicrobial (*Candidatus* Methylacidiphilum) aerobic methanotrophs together with anaerobic methanotrophs: ‘Candidatus Methanoperedens nitroreducens’ and NC10 phylum bacteria based on 16S rRNA gene amplification. Reads were assigned to phylogenetic groups based on the SILVA NGS pipeline. The soil depth in centimeters is depicted vertically, and the total amount of 16S rRNA gene amplicons per gram dry weight is depicted horizontally. The colored bars represent the relative amount of gene copies corresponding to aerobic and anaerobic methanotrophs present in the soil at a particular depth.

At each depth, the 16S rRNA gene amplicon data were analyzed for both bacteria and archaea. In the bacterial community, a very large diversity was observed (**Figure 3** and Supplementary Table S1), with most of the reads assigned to *Acidobacteria, Chloroflexi, Proteobacteria, Planctomycetes,* and *Actinobacteria*. Most of the phyla were observed throughout the soil core. However, at depths of 40 cm and below, *Cyanobacteria*, *Bacteroidetes,* and *Chlorobi* were hardly or not present at all. The opposite trend was observed for *Latescibacteria*, which increased gradually in relative abundance toward deeper layers. 16S rRNA gene reads assigned to NC10 phylum bacteria (classified into phylum *Nitrospirae* by Silva NGS) were recorded at all depths along the gradient of the soil core. The lowest relative abundance was recorded at the top layer of soil (0 cm). Thereafter, the copies increased gradually, with a maximum at a depth of 40 cm, where reads assigned to the NC10 phylum represented 2.4% of the total bacterial 16S rRNA gene reads. After 40 cm, a rapid decrease was observed in the relative abundance of reads assigned to the NC10 phylum to 50 cm (0.25%), followed by an increase at 60 cm (1.25%). The relative abundance of reads assigned to the NC10 phylum at all other depths, except 35 cm, 40 cm, and 60 cm, was less than 1% of the total bacterial reads (**Figure 4**).

Among *Proteobacteria*, the relative abundance of *Alphaproteobacteria* was highest in the top 15 cm of the soil core and gradually decreased in the deeper layers of soil. *Beta*- and *Gammaproteobacteria* showed the lowest relative abundances, but their relative abundances exhibited little variation throughout the soil core. *Deltaproteobacteria* were the second most abundant in the top 15 cm. Their relative abundance peaked at 25 cm, corresponding to 8% of total bacterial reads, and decreased gradually thereafter. A detailed distribution of the proteobacterial classes is provided in Supplementary Table S2.

Among sequences assigned to aerobic methanotrophs, most of the reads were assigned to *Methylococcaceae*, except at depths of 0 cm and 15 cm, where more reads were assigned to *Methylocystaceae*. Surprisingly, we observed *Verrucomicrobia* methanotrophs in the paddy soil core, and reads assigned to *Candidatus* Methylacidiphilum were most abundant in the top 5 cm, constituting 20% of the total aerobic methanotrophic community. Overall, the relative abundance of aerobic methanotrophs was highest at a depth of 10 cm, representing 1.1% of the total bacterial community. The calculated abundance of aerobic methanotrophs in calculated copy numbers was on the order of 10^7^ in the top 20 cm and then gradually decreased to 10^4^ at a depth of 60 cm (**Figure 4**).

### Phylogenetic Diversity of *Verrucomicrobia* and *Candidatus* Methylacidiphilum

**FIGURE 5:**
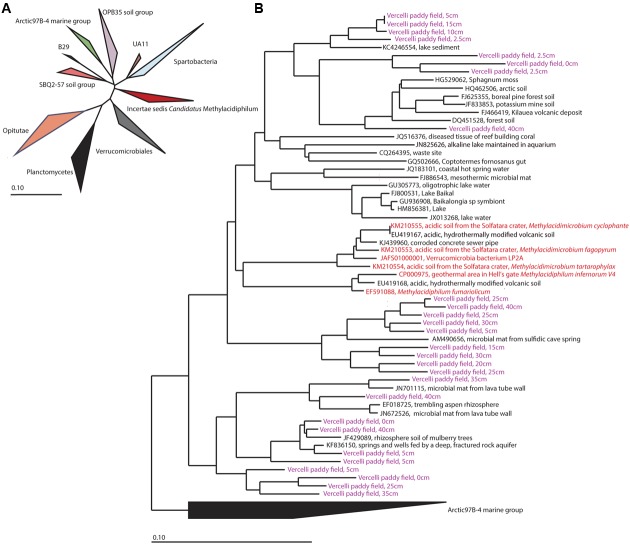
**(A)** Phylogenetic overview of verrucomicrobial 16S rRNA gene sequences. The phylogenetic position of *Candidatus* Methylacidiphilum (*Incertae sedis Candidatus* Methylacidiphilum) is marked in red. **(B)** Detailed presentation of the sequences of *Candidatus* Methylacidiphilum. Sequences of cultivated strains are shown in red, and sequences obtained from paddy field soil are shown in purple. The neighbor-joining phylogenetic tree was calculated using Jukes-Cantor correction and the Arctic 97B-4 marine group as outgroup.

Verrucomicrobial methanotrophs have rarely been observed outside acidic volcanic areas. Therefore, we extracted the 16S rRNA gene sequences from amplicon sequencing and analyzed sequences assigned to *Verrucomicrobia* in detail (**Figure 5**). Sequences clustering with *Candidatus* Methylacidiphilum were found at all depths except 7.5 cm, 50 cm, and 60 cm. The extracted sequences clustering with *Candidatus* Methylacidiphilum were 85% identical at the nucleotide level to cultivated strains of *Candidatus* Methylacidiphilum (van Teeseling et al., 2014).

### Amplicon Sequencing of the 16S rRNA Gene of the Archaeal Community

**FIGURE 6:**
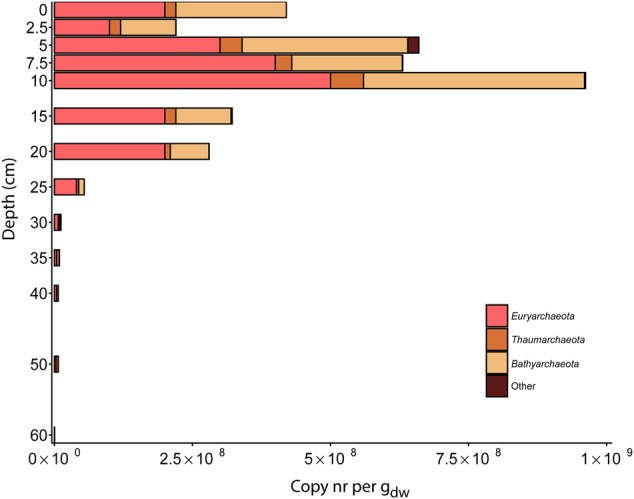
Distribution of 16S rRNA gene reads of major archaeal phyla along the depth profile of the paddy soil core. Reads were assigned to phylogenetic groups based on the SILVA NGS pipeline. The soil depth in centimeters is depicted vertically, and the total amount of 16S rRNA gene amplicons per gram dry weight is depicted horizontally. The colored bars represent the relative amount of gene copies matching an archaeal phylum present in the soil at a particular depth.

In the archaeal community reads matching *Euryarchaeota* were more abundant than *Thaumarchaeota* in the top layers until a depth of 30 cm. At deeper depths of 35 cm, 50 cm, and 60 cm (*Euryarchaeota* were dominant at 40 cm), sequences matching *Thaumarchaeota* were the most abundant. Sequences matching the 16S rRNA gene of *Bathyarchaeota* (previously known as *Miscellaneous Crenarchaeota Group* (MCG)) were the most abundant in the top layers of the soil. At depths of 0–5 cm, 43–45% of the reads were assigned as *Bathyarchaeota*. This proportion decreased gradually throughout the soil core (**Figure 6** and Supplementary Table S3).

**FIGURE 7:**
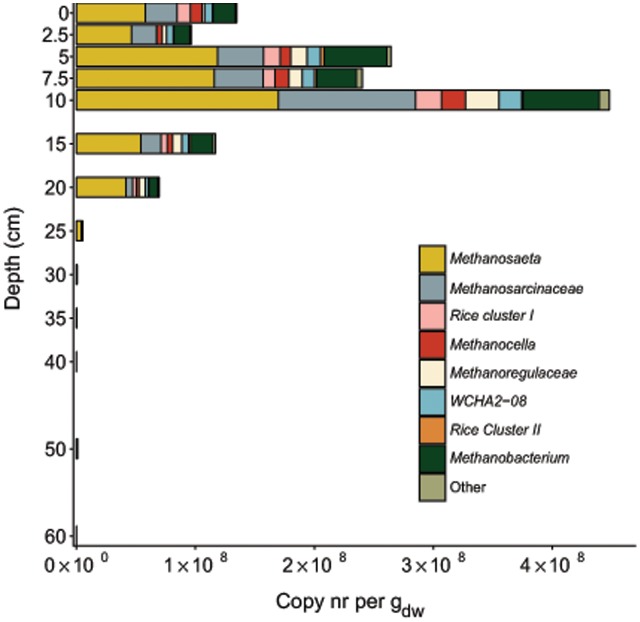
Distribution of 16S rRNA gene reads of methanogens along the depth profile of the paddy soil core. Reads were assigned to phylogenetic groups based on the SILVA NGS pipeline. The soil depth in centimeters is depicted vertically, and the total amount of 16S rRNA gene amplicons per gram dry weight is depicted horizontally. The colored bars represent the relative amount of gene copies matching methanogens present in the soil at a particular depth.

Analysis of the *Methanomicrobia* and *Methanobacteria* communities in greater detail revealed that sequencing reads assigned to *Methanosaeta* and *Methanosarcina* were most abundant among methanogens throughout the soil core (**Figure 7**). The top layer of soil had more diverse community than deeper layers. The methanogen community was largest at a depth of 10 cm, 52% of total archaea. The highest relative sequence abundance of the archaeal methanotroph ‘*Candidatus* Methanoperedens nitroreducens’ (GOM Arc I) was found at a depth of 25 cm, comprising 56.4% of the total archaeal reads. The estimated depth distribution of ‘*Candidatus* Methanoperedens nitroreducens’ calculated based on the sequencing read abundance and on the total archaeal copy numbers is depicted in **Figure 4**. The abundance based on amplicon data peaked at 1.1^∗^10^8^ copies at a depth of 7.5 cm.

### Soil Slurry Incubations

**FIGURE 8:**
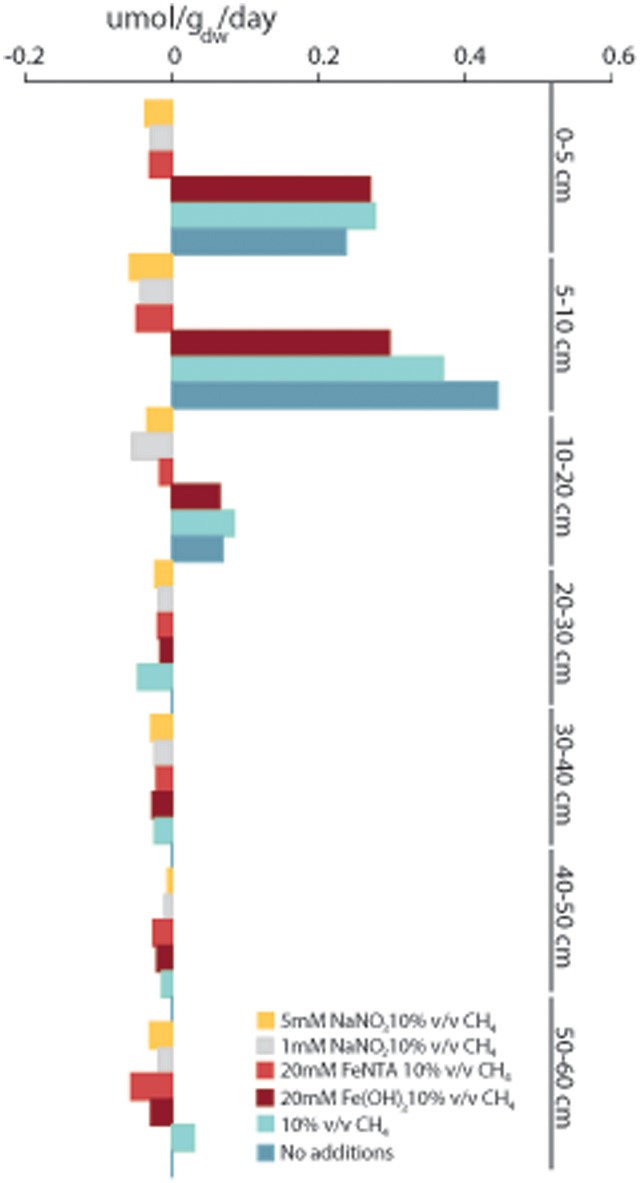
Methane oxidation and methanogenesis rates measured in soil slurries incubated with 10% v/v 13C-methane with the addition of 5 mM NaNO3, 1 mM NaNO2, 20 mM FeNTA or 20 mM ferrihydrite. As controls, soil slurries were incubated with 10% v/v 13C-methane and without added methane. GC measurements were performed in triplicate, and rates were calculated in the linear phase. Negative values stand for net oxidation of methane and positive values net production of methane.

Soil slurries of different depths were amended with methane and electron acceptors. Controls were prepared with and without addition of methane to detect methanogenic activity. The rates of potential methane oxidation with nitrate, nitrite and two forms of iron, FeNTA and ferrihydrite, were recorded. The potential methane oxidation or production rate was calculated based on methane concentration measurements in the headspace over a time course of 118 days (**Figure 8**).

The nitrate- and nitrite-dependent methane oxidation rates were highest in the top 20 cm. At depths of (0–5 cm) and (5–10 cm), higher AOM rates were measured in slurries amended with nitrate, 37 nmol g_dw_ h^-1^ and 57 nmol g_dw_ h^-1^, than in slurries amended with nitrite, 29 nmol g_dw_ h^-1^ and 43 nmol g_dw_ h^-1^, respectively. At a depth of 10–20 cm, the nitrite-amended samples exhibited the highest methane oxidation rate, 55 nmol g_dw_ h^-1^, followed by 33 nmol g_dw_ h^-1^ in the nitrate-amended samples. Methane oxidation was measured in the top 20-cm slurries in samples amended with FeNTA, with a peak of 48 nmol g_dw_ h^-1^ at a depth of 5–10 cm.

In the top layers up to 20 cm, addition of ferrihydrite did not stimulate methane oxidation. In the deeper layers, the pattern was the same as that for the addition of FeNTA. The highest methane oxidation rate was observed at a depth of 40–50 cm in slurries amended with FeNTA, 25 nmol g_dw_ h^-1^, followed by ferrihydrite, 20 nmol g_dw_ h^-1^. At a depth of 50–60 cm, the respective rates for FeNTA and ferrihydrite were 56 nmol g_dw_ h^-1^ and 29 nmol g_dw_ h^-1^.

In the control samples amended with methane, initial methane oxidation was monitored for a maximum time period of 21 days, after which methane production prevailed, with production of 277 nmol g_dw_ h^-1^, 369 nmol g_dw_ h^-1^ and 85 nmol g_dw_ h^-1^ at depths of 0–5 cm, 5–10 cm, and 10–20 cm, respectively. A similar pattern of methanogenesis in soil slurry incubations with no additions was observed. After a lag phase of approximately 21 days, the methane production rate increased. The highest methane production rate, 444 nmol g_dw_ h^-1^, was observed at a depth of 5–10 cm. At a depth of 10–20 cm, methanogenesis was still observed, with a rate of 69 nmol g_dw_ h^-1^, which decreased to less than 1 nmol g_dw_ h^-1^ in deeper layers. At depths of 20–50 cm, oxidation prevailed over methane production in slurries amended with methane.

## Discussion

Paddy fields are a major source of methane emitted to the atmosphere. The flux of methane is controlled by the microbial community present in the soil, particularly by methanogens and methanotrophs.

The vertical profile of the methane gradient included a higher methane concentration in the top 15 cm of the soil core, followed by a drastic drop. At a depth of approximately 28 cm, methane was nearly undetectable. This depth correlates with the interface of annual plowing and undisturbed soil as well as the rice root penetration depth.

The profile of the total abundance of microorganisms along the depth gradient followed the same trend as methane. The highest copy numbers of both bacteria and archaea were detected at a depth of 10 cm, followed by a decrease to 25 cm, after which the microbial population size was a few orders of magnitude smaller. The total bacterial and archaeal population sizes correlate well with previous reports. The total bacterial and archaeal 16S rRNA gene copy numbers in a Chinese paddy field ranged from 1.4^∗^10^10^ to 2.9^∗^10^10^ per g_dw_ and 5.4^∗^10^8^-1.7^∗^10^9^ per g_dw_, respectively (Ahn et al., 2012). In paddy fields in the Philippines, the total bacterial copy numbers and archaeal copy numbers were on the order of 10^10^ and 10^8^ per g_dw_, respectively (Breidenbach and Conrad, 2015). In paddy fields in India, 9.6^∗^10^9^-1.4^∗^10^10^ bacterial 16S rRNA copies per g_dw_ and 7.13^∗^10^7^-3.02^∗^10^8^ archaeal 16S rRNA copies per g_dw_ were reported (Singh et al., 2012). We recorded maximum bacterial and archaeal abundances of 5.6 ± 1.4^∗^10^9^ and 1.0 ± 0.3^∗^10^9^ 16S rRNA gene copies per g_dw_, respectively.

The rice root system has been described as the key determinant in shaping the microbial community via release of root exudates, decaying roots and organic matter as well as oxygen (Kuzyakov and Blagodatskaya, 2015). Diffusion of oxygen to the soil creates micro-oxic niches for oxygen-dependent microorganisms. We detected sequences belonging to aerobic methanotrophs throughout the soil core. The relative abundance of aerobic methanotrophs was highest at a depth of 10 cm and was twice as high as of that in the surface layer. Along the entire depth gradient, MOB were dominated by Type I *Methylococcaceae*, followed by Type II *Methylocystaceae*. *Methylococcaceae* have been detected in several environments with low oxygen concentration, even tolerating periods of hypoxia (Hernandez et al., 2015). The presence of these aerobic methanotrophs in low oxygen environments, such as the investigated paddy field, could possibly be explained by their denitrifying ability as has been demonstrated for *Methylomonas denitrificans*, which during hypoxia carries out nitrate reduction and methane oxidation (Kits et al., 2015). Other *Methylococcaceae*, such as *Methylobacter* contain in their genome besides respiratory nitrate and nitrite reductases as well genes necessary for dinitrogen fixation (Kalyuzhnaya et al., 2015).

In addition to detecting sequences of well-known proteobacterial aerobic methane oxidizers, sequences belonging to methanotrophic *Verrucomicrobia* were identified in this study. Detailed phylogenetic analysis revealed the presence of aerobic methanotrophs distantly related (85% nucleotide identity of the 16S rRNA gene) to cultured members of *Candidatus* Methylacidiphilum. Only a very small number of verrucomicrobial methanotrophs have been detected in ecosystems other than acidic volcanic areas, including paddy field soil (GenBank JF984005.1), forest soil (GenBank JF420089), lake sediment (GenBank GU305773) arctic soil (GenBank HQ462506) and a few other environments. The reported cultivated strains originate exclusively from extreme hot or acidic environments in Italy, Kamchatka or New Zealand. Further studies are needed to determine if the microbes found in less extreme environments also contain *pmoA* genes in their genome and have the capability to oxidize methane. We hypothesize that there is a niche for these aerobic verrucomicrobial methane oxidizers in less acidic methane-rich environments such as paddy fields.

The translation of 16S rRNA gene sequencing read numbers to copy numbers indicated that the methanogen population abundance was highest at a depth of 10 cm, with 4.5^∗^10^8^ copies per g_dw_, followed by a decline in abundance to 60 cm, with 2.7^∗^10^4^ copies per g_dw_. The methanogenic population size determined previously in the same Italian paddy field was 10^7^–10^8^ copies per g_dw_ (Conrad and Klose, 2006). Compared to other sampling sites, our observed abundances are slightly higher than the previously reported methanogen abundances of 1.1^∗^10^7^ or 1.4^∗^10^7^ copies per g_dw_ (Singh et al., 2012) or 10^4^–10^5^ copies per g_dw_ (Hou et al., 2000). A previous vertical profile study of methanogens identified the highest abundance based on *mcrA* gene copy numbers at a depth of approximately 20 cm in three Japanese paddy fields, peaking at 10^7^ (Watanabe et al., 2010). Together, these results suggest that the methanogenic zone is located approximately 10–20 cm below the soil surface and co-occurs with the end of the main root system in soil.

The community analysis of methanogens revealed a diverse composition throughout the soil core. The methanogenic community was dominated by *Methanosaeta, Methanosarcina, Methanobacterium, Methanoregulaceae,* and the RC I cluster (*Methanocella*), which have also been found previously in temperate climate paddy fields (Conrad and Klose, 2006; Watanabe et al., 2010). The community throughout the core was dominated by the strictly acetoclastic *Methanosaeta*, followed by more versatile *Methanosarcina* spp. The sampling time of the soil at the end of the growing season, when most root exudates are released (Aulakh et al., 2001) and the ammonia concentration is highest, may explain the methanogen community structure (Singh et al., 2012). *Methanosarcina* spp. have been shown to be present during the rice-growing season, whereas during pre-planting, tilling or post-harvest, *Methanosaeta* were present in lower numbers (Singh et al., 2012), correlating with the lower concentrations of acetate available in the soil (Kruger et al., 2002). In paddy field soil, acetate-dependent methanogenesis (acetoclastic) generally dominates over hydrogen-dependent methanogenesis (hydrogenotrophic), as demonstrated by ^13^C-labeling experiments (Conrad, 1999, 2005; Conrad et al., 2002; Zhang et al., 2016).

The total methane concentration in soil over the course of rice maturation peaks at the flowering and ripening stage (Singh et al., 2012). Previous studies in Italian paddy fields have demonstrated that methane emission rates reach approximately 400 nmol CH_4_ per g_dw_ d^-1^ 70–80 days after flooding (Kruger et al., 2005) or even approximately 600 nmol per g_dw_ d^-1^ (Conrad and Klose, 2006). We previously observed methanogenic activity of the same paddy field soil of 432 nmol and 358 nmol per g_dw_ d^-1^ without and with the addition of methane, respectively, in incubation assays (Vaksmaa et al., 2016). In the current soil core, the highest methanogenic activity was recorded at a depth of 5–10 cm, with rates of 369 and 444 nmol per g_dw_ d^-1^ with and without the addition of methane. In control incubations in which methane was added, methanotrophic activity was initially observed. After 3 weeks, methanogenesis became the dominant process, with methane oxidation rates identical to those observed in the control treatment without the addition of methane. Furthermore, ferrihydrite added to slurry incubations seemed to stimulate methanogens in the top 20 cm. In those treatments, no initial methanotrophic activity was observed; only stimulation of methanogenic activity was recorded, even when both methane and possible electron donors were supplied. Previous studies in wetlands and paddy fields have demonstrated that the addition of poorly crystalline iron, such as ferrihydrite, has an inhibitory effect on methanogenic activity (Achtnich et al., 1995; Lueders and Friedrich, 2002). In contrast, the addition of highly crystalline iron oxide species of hematite or magnetite stimulates methanogens enriched from paddy field soil via a positive effect on either direct interspecies electron transfer or the availability of diffusive carriers such as hydrogen or formic acid (Kato et al., 2012; Holmes et al., 2017).

Methanotrophic bacteria of the NC10 phylum have been previously detected in paddy field soil based on the 16S rRNA gene or *pmoA* genes and activity assays with nitrite and methane. Conflicting results regarding the vertical distribution of NC10 phylum bacteria in soil have been reported. Zhou et al. (2014) indicated that the highest abundance of 1.0^∗^10^8^ copies per g_dw_ occurred at a depth of 100–120 cm (Zhou et al., 2014). This finding was supported by a study by Hu et al. (2014) of a Chinese paddy field, in which the highest copy number abundance of 1.5 ± 0.2^∗^10^6^ to 4.5 ± 0.3^∗^10^6^ copies per g_dw_ was observed at a depth of 50–60 cm. However, the methane-oxidizing potential of soil slurries amended with nitrite was highest at a depth of 90–100 cm, with values of 1.68 ± 0.03 to 2.04 ± 0.06 nmol of CO_2_ per g_dw_ (Hu et al., 2014). By contrast, in a subtropical paddy field soil core sampled to 100 cm, the abundance of NC10 phylum bacteria was highest at the 0–10 cm depth, with 1.0 ± 0.1^∗^10^5^ copies per g_dw_, followed by 7.5 ± 0.4^∗^10^4^ at 30–40 cm and a subsequent gradual decrease, with no detection at depths of 70 cm and beyond (Wang et al., 2012).

The phylogenetic comparison of the 16S rRNA gene reads obtained from amplicon sequencing revealed that, in the top layers, the 16S rRNA gene reads were assigned exclusively to group B (Ettwig et al., 2009; Welte et al., 2016), with nucleotide identities of 95.6–96.7% to *‘Candidatus* Methylomirabilis oxyfera.’ Sequences belonging to Group A of NC10 phylum bacteria were found only at depths of 40 cm and below. This distribution is consistent with previous reports in which 16S rRNA gene sequencing and relative read abundance indicated that these nitrite-dependent AOM bacteria formed the largest subset of sequencing reads among total bacterial reads at depths of 50 cm and 100 cm (Ding et al., 2015). In our activity assays with nitrite, we observed the highest methane oxidation potential of 55 nmol per g_dw_ h^-1^ in samples from 10 to 20 cm, which correlates with the first peak of high abundance of NC10 phylum bacteria. However, all the sequences at 10–20 cm all belonged to group B, for which no methane-oxidizing ability has been demonstrated thus far and needs further investigation.

In addition to detecting nitrite-dependent AOM bacteria of the NC10 phylum, we observed high numbers nitrate-dependent AOM archaea ‘*Candidatus* Methanoperedens nitroreducens’ throughout the soil core. However compared to NC10 phylum bacteria, these archaea were more abundant at all depths, except 60 cm, where NC10 phylum bacteria outnumbered ‘*Candidatus* Methanoperedens nitroreducens.’ Sequences classified as ‘*Candidatus* Methanoperedens nitroreducens’ have been detected previously in paddy fields, including fields in Vercelli, Italy (Lueders et al., 2001; Conrad et al., 2008), Chinese paddy fields (Xu et al., 2012), and Korean paddy fields (Lee et al., 2015) as well as in natural wetlands (Narrowe et al., 2017). We previously quantified and detected ‘*Candidatus* Methanoperedens nitroreducens’ in an Italian paddy field based on 16S rRNA gene (Vaksmaa et al., 2016) and *mcrA* gene sequences in high abundance (Vaksmaa et al., 2017a). High relative sequence abundance has also been observed in other paddy fields based on the 16S rRNA gene, with 60% of all archaeal reads classified as ‘*Candidatus* Methanoperedens nitroreducens’ (GOM Arc I) at a depth of 60 cm in bulk soil (Lee et al., 2015). In a study by Lee et al. (2015), the soil core depth profile exhibited the same trend observed in the current study (Lee et al., 2015). The abundance of ‘*Candidatus* Methanoperedens nitroreducens’ increased with depth, peaking at 20 cm with 1.8 ± 0.3^∗^10^7^copies per g_dw_. The activity assays performed with nitrate and methane indicated that the activity was highest at a depth of 5–10 cm (57 nmol per g_dw_ d^-1^), followed by a depth of 10–20 cm (33 nmol per g_dw_ d^-1^). We previously observed a methane-oxidizing potential of 80 nmol methane per g_dw_ d^-1^ in mixed and sieved soil slurry from a depth of 10–20 cm (Vaksmaa et al., 2016). The present study is the first to evaluate potential methane oxidation rates utilizing nitrate as an electron acceptor in different depths of a paddy soil core. Recent research has revealed that ‘*Candidatus* Methanoperedens nitroreducens’ not only can couple nitrate reduction to methane oxidation but is also able to reduce oxidized metals (Ettwig et al., 2016) and may play in important role in both methane and iron cycling in natural and man-made wetlands (Narrowe et al., 2017).

Finally the large number of *Bathyarchaeota* observed in this paddy field and other wetland systems is intriguing, and their potential role in methane cycling needs further investigation (Evans et al., 2015; Narrowe et al., 2017). For the microbial community members, which the function is still unknown, we detected *Bathyarchaeota* to be present throughout soil core with highest abundance at 5 cm with 2.1 ± 1.1^∗^10^8^ 16S rRNA gene copies per g_dw._ From the total archaeal community, these account for almost 50%. Albeit their unknown function, their high abundance and wide distribution indicates that though the function is unknown they might be relevant microorganisms. Up to date, the members of phylum *Bathyarchaeota* have been detected in a wide range of habitats from terrestrial to marine, cold and hot temperatures or surface and subsurface environments. Generally they are known to be abundant in marine environments (Teske et al., 2002; Lipp et al., 2008; Kubo et al., 2012). Similarly, other studies showed *Bathyarchaeota* to be present in freshwater environments (Porat et al., 2010; Li et al., 2012). Previous studies have detected *Bathyarchaeota* in paddy fields as well (Lee et al., 2014, 2015) with abundance of *Bathyarchaeota* increasing from 17 to 23% in three different phases of rice cultivation (Breidenbach and Conrad, 2015) and with relative abundance up to 42% in paddy field sub-soils (Bai et al., 2017).

In summary, we observed high diversity of the archaeal and bacterial microbial communities throughout the soil core and determined the methane-oxidation potential with various electron acceptors at several soil depths. This study highlights the usage of various electron acceptors for the AOM process. Our findings provide support for the significant role of ‘*Candidatus* Methanoperedens nitroreducens’ carrying out nitrate-dependent and/or iron-dependent AOM in paddy fields. NC10 phylum bacteria seem to play a less significant role in AOM in paddy fields. The as-yet unknown functions of members of the *Candidatus* Methylacidiphilum genus and *Bathyarchaeota* in paddy field soil will hopefully be explained in studies in the near future. We acknowledge that the small sample size of our study does have its limitations, and future studies should include more samples in order to more accurately estimate the contribution of AOM in paddy fields on a larger scale.

## Author Contributions

AV and CL designed the research and carried out the fieldwork. EL and GV provided the access to the sampling station, and supported the design and fieldwork. AV carried out the experiments in the laboratory. TvA and AV collected and interpreted the sequencing data. AV, CL, KE, and MJ drafted and finalized the manuscript with input from all authors. The manuscript was checked by a professional peerwith.com editor.

## Conflict of Interest Statement

The authors declare that the research was conducted in the absence of any commercial or financial relationships that could be construed as a potential conflict of interest.

## Footnotes

1 http://www.bioinformatics.org/cd-hit

2 http://www.arb-silva.de

3 http://blast.ncbi.nlm.nih.gov/Blast.cgi
