# Supplementary material for: Stratification of Diversity and Activity of Methanogenic and Methanotrophic Microorganisms in a Nitrogen-Fertilized Italian Paddy Soil
Source: Front Microbiol. 2017 Nov 13;8:2127. doi: 10.3389/fmicb.2017.02127 (PMC5693880; doi:10.3389/fmicb.2017.02127)
Supplement: Supplementary file 1 [file Table_1.doc]

***Supplementary Material***

**Stratification of diversity and activity of methanogenic and methanotrophic microorganisms in a nitrogen-fertilized Italian paddy soil**

A. Vaksmaa^1^, T. A. van Alen^1^, K.F. Ettwig^1^, E. Lupotto^2^, G. Valè^2^, M. S. M. Jetten^1^ & C. Lüke^1^

**Correspondence:** Annika Vaksmaa, a.vaksmaa@science.ru.nl

Table S1: Distribution of sequence reads from Bacterial 16S rRNA gene amplicon. Reads were assigned to phylogenetic groups based on the SILVA NGS pipeline. Values are expressed as percentage of total reads of Bacteria.

| Depth (cm) | *Acidobacteria* | *Actinobacteria* | *Bacteroidetes* | *Chloroflexi* | *Cyanobacteria* | *Firmicutes* | *Gemmatimonadetes* | *Verrucomicrobia* | *Nitrospirae* | *Planctomycetes* | *Proteobacteria* | *Parcubacteria* | *Chlorobi* | *Latescibacteria* | *Other* |
| --- | --- | --- | --- | --- | --- | --- | --- | --- | --- | --- | --- | --- | --- | --- | --- |
| 0 | 17.70 | 9.95 | 2.54 | 21.58 | 1.69 | 1.78 | 2.39 | 2.85 | 1.77 | 9.70 | 16.82 | 5.35 | 1.36 | 1.03 | 3.48 |
| 2.5 | 17.64 | 9.28 | 3.08 | 21.58 | 0.74 | 1.89 | 2.47 | 3.01 | 2.27 | 7.91 | 18.81 | 4.98 | 1.77 | 1.61 | 2.96 |
| 5 | 17.25 | 7.09 | 2.60 | 21.42 | 1.13 | 1.48 | 1.95 | 4.16 | 2.92 | 9.40 | 19.05 | 4.35 | 1.29 | 1.99 | 3.91 |
| 7.5 | 14.89 | 7.90 | 2.67 | 24.55 | 1.16 | 1.48 | 2.25 | 4.13 | 2.37 | 10.01 | 17.07 | 4.35 | 1.46 | 1.98 | 3.73 |
| 10 | 16.08 | 8.01 | 2.72 | 20.76 | 0.84 | 1.90 | 2.58 | 3.19 | 2.06 | 10.21 | 18.73 | 5.10 | 1.50 | 2.07 | 4.25 |
| 15 | 19.31 | 7.61 | 2.24 | 22.30 | 0.61 | 2.01 | 1.84 | 4.01 | 2.13 | 12.77 | 15.25 | 2.78 | 1.05 | 2.40 | 3.71 |
| 20 | 16.01 | 4.66 | 1.89 | 26.68 | 0.43 | 2.03 | 3.08 | 2.51 | 3.31 | 11.73 | 15.28 | 3.60 | 1.61 | 3.00 | 4.18 |
| 25 | 18.92 | 3.04 | 1.19 | 20.68 | 0.09 | 1.92 | 2.66 | 2.52 | 4.57 | 11.26 | 15.37 | 7.91 | 1.61 | 3.75 | 4.51 |
| 30 | 16.07 | 5.11 | 0.93 | 19.34 | 0.22 | 2.44 | 3.12 | 2.36 | 8.23 | 11.79 | 14.22 | 5.65 | 1.02 | 4.02 | 5.48 |
| 35 | 17.76 | 4.24 | 0.14 | 26.08 | 0.03 | 0.33 | 3.86 | 0.89 | 10.55 | 13.12 | 8.51 | 4.05 | 0.73 | 4.72 | 4.97 |
| 40 | 17.64 | 3.64 | 0.21 | 21.28 | 0.06 | 2.49 | 3.43 | 2.03 | 11.65 | 13.26 | 10.31 | 3.72 | 0.50 | 5.77 | 4.00 |
| 50 | 22.74 | 7.03 | 0.34 | 24.64 | 0.11 | 0.55 | 1.56 | 1.91 | 6.25 | 10.18 | 11.84 | 3.63 | 0.53 | 5.10 | 3.56 |
| 60 | 17.35 | 13.33 | 0.55 | 22.59 | 0.23 | 0.82 | 2.94 | 2.39 | 6.98 | 8.96 | 13.63 | 2.30 | 0.41 | 3.74 | 3.78 |
