# Supplementary material for: Stratification of Diversity and Activity of Methanogenic and Methanotrophic Microorganisms in a Nitrogen-Fertilized Italian Paddy Soil
Source: Front Microbiol. 2017 Nov 13;8:2127. doi: 10.3389/fmicb.2017.02127 (PMC5693880; doi:10.3389/fmicb.2017.02127)
Supplement: Supplementary file 2 [file Table_2.doc]

***Supplementary Material***

**Stratification of diversity and activity of methanogenic and methanotrophic microorganisms in a nitrogen-fertilized Italian paddy soil**

A. Vaksmaa^1^, T. A. van Alen^1^, K.F. Ettwig^1^, E. Lupotto^2^, G. Valè^2^, M. S. M. Jetten^1^ & C. Lüke^1^

**Correspondence:** Annika Vaksmaa, a.vaksmaa@science.ru.nl

Table S2: Distribution of sequence reads of proteobacteria. Reads were assigned to phylogenetic groups based on the SILVA NGS pipeline. Values are expressed as percentage of total reads of Bacteria.

| Depth (cm) | Alpha-proteobacteria | Beta-proteobacteria | Delta-proteobacteria | Gamma-proteobacteria |
| --- | --- | --- | --- | --- |
| 0 | 8..11 | 2.56 | 4.01 | 1.47 |
| 2.5 | 9.79 | 3.81 | 6.97 | 2.12 |
| 5 | 9.25 | 4.73 | 7.45 | 2.36 |
| 7.5 | 7.86 | 3.33 | 5,45 | 1.75 |
| 10 | 9.44 | 4.40 | 7.62 | 3.29 |
| 15 | 6.33 | 3.11 | 5.23 | 1.86 |
| 20 | 5.15 | 2.69 | 5.96 | 1.55 |
| 25 | 4.31 | 4.27 | 8.02 | 1.45 |
| 30 | 2.43 | 2.41 | 4.62 | 1.09 |
| 35 | 2.45 | 1.99 | 4.88 | 0.39 |
| 40 | 3.15 | 2.72 | 2.61 | 0.52 |
| 50 | 2.83 | 3.86 | 2.02 | 0.61 |
| 60 | 2.98 | 4.29 | 2.76 | 0.74 |
